# Safety and efficacy of *Qishen* granules in patients with chronic heart failure: study protocol for a randomized controlled trial

**DOI:** 10.1186/s13063-017-2193-z

**Published:** 2017-10-10

**Authors:** Jinping Wang, Jun Shi, Jiawei Wei, Juan Wang, Kuo Gao, Xueli Li, Jianxin Chen, Shaojing Li, Huihui Zhao, Wei Wang

**Affiliations:** 1 Beijing University of Chinese Medicine, Bei San Huan Dong Lu, Chao Yang District, Beijing, 100029 China; 2 Department of Physiology, Basic Medical College, Inner Mongolia Medical University, Xinhua Street, Hui Min district, Hohhot, China; 3 China Academy of Chinese Medical Sciences Institute of Chinese Materia Medica, Beijing, China; 4 Beijing Key Laboratory of Syndrome and Prescription Basic Research, Beijing University of Chinese Medicine, Beijing, 100029 China

**Keywords:** Chronic heart failure, *Qishen* granules, Efficacy, Safety, Traditional Chinese herbal medicine

## Abstract

**Background:**

Chronic heart failure (CHF), the final stage of various cardiovascular diseases, is a major public health problem resulting in significant hospitalization rates, mortality, and huge health care costs despite advances in the treatment and management of heart failure and heart failure-related risk factors. *Qishen* granules (QSG), a Chinese herbal formula, is widely used by traditional Chinese medicine (TCM) practitioners to treat CHF. Several animal experimental studies have showed that QSG can significantly relieve the heart failure symptoms in CHF rat models. However, there is as yet no standard clinical trial to confirm this. Thus, the investigators are conducting this study to evaluate the efficacy and safety of QSG in a large, and varied population.

**Methods/design:**

This study is designed as a randomized, placebo-controlled, multi-center, double-blind clinical trial with parallel groups. A total of 200 patients with CHF will be recruited and randomly allocated to either the QSG treatment group or the placebo group (in a 1:1 ratio). The patients will receive QSG or placebo granules twice a day for 12 weeks. The primary outcome is the proportion of patients in the QSG group, compared with the placebo group, demonstrating a more than 30% decrease in NT-proBNP level during 12 weeks of treatment. The secondary outcomes consist of composite cardiac events, New York Heart Association functional classification, 6-minute walking distance, left ventricular ejection fraction, patient quality of life, and the TCM syndrome integral scale.

**Discussion:**

On a background of standard treatment, QSG may further reduce the levels of NT-proBNP. This trial will provide high-quality evidence on the efficacy and safety of QSG in treating CHF, thus providing reference for clinical application of QSG.

**Trial registration:**

Clinical Trials.gov: NCT03027375. Registered on 16 January 2017.

**Electronic supplementary material:**

The online version of this article (doi:10.1186/s13063-017-2193-z) contains supplementary material, which is available to authorized users.

## Background

Chronic heart failure (CHF), the final stage of various myocardial diseases, is a chronic clinical syndrome that occurs when the heart is unable to pump blood within the circulatory system and organs cannot receive sufficient oxygen and nutrients [1]. Evidence from the American Heart Association’s report has demonstrated that the 2013 overall death rate attributable to cardiovascular disease was 222.9 per 100,000 Americans, of which 269.8 were men and 184.8 were women [2]. Meanwhile, despite advances in the treatment strategy for heart failure, such as evidence-based approaches to treat heart failure risk factors and implementation of angiotensin-converting enzyme inhibitors, β-blockers, coronary revascularization, implantable cardioverter-defibrillators, and cardiac resynchronization therapeutic strategies, the outcome often remains unsatisfactory. The most recent data from an international congestive heart failure prospective cohort study demonstrated that overall mortality within 1 year was 16.5% [3].

Traditional Chinese medicine (TCM), with a much lower cost and a significant curative effect on CHF-related symptoms, has been widely used in the treatment of CHF in China [4]. From the perspective of TCM, the primary cause of heart failure is heart *Qi* deficiency and blood stasis. *Qishen* granules (QSG) are prepared from a composition of six TCM herbs, including *Astragalus membranaceus* (Fisch.) Bge. var. *mongolicus* (Bge.) Hsiao, *Salvia miltiorrhiza* Bge., *Lonicera japonica* Thunb., *Scrophularia ningpoensis* Hemsl., *Aconitum carmichaeli* Debx., and *Glycyrrhiza uralensis* Fisch. This formula is widely used to treat the CHF of *Qi* deficiency and the blood stasis syndrome. Several animal experimental studies have showed the effect of QSG on the heart failure symptoms in CHF rat models [5–9], but there is yet no standard clinical evidence. Therefore, the investigators have designed a clinical study to assess the efficacy and safety of QSG in a large and varied population.

## Methods/design

### Objective

The objective of this study is to evaluate the safety and efficacy of QSG in a large and varied CHF population.

### Design

**Fig. 1:**
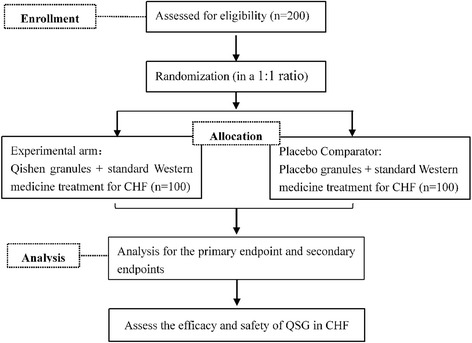
Flowchart of this study

This study is designed as a randomized, placebo-controlled, double-blind, parallel-group, multi-center study in three tertiary academic medical centers of China to evaluate the safety and efficacy of QSG. The flowchart is represented in Fig. 1. The Standard Protocol Items: Recommendations for Interventional Trials (SPIRIT) Checklist can be found in Additional file 1.

Patient enrollment is expected to last up to 12 months. The end of the study is defined by the final follow-up of the last enrolled patient. This trial has been registered at ClinicalTrials.gov (ID: NCT03027375).

Qualified patients will be randomized into either of the two groups: the QSG treatment group or the placebo group (in a 1:1 ratio). Each group will receive either QSG or placebo granules in addition to standard care and appropriate medical support prescribed for CHF, which will be prescribed by the attending physicians based on the Chinese guidelines published in 2014 for the diagnosis and treatment of heart failure [10].

Recruited patients will receive QSG or placebo granules twice a day for 12 weeks. The QSG and placebo used in this study will be manufactured in China in accordance with the China Pharmacopoeia standard of quality control.

### Setting

There are three tertiary, academic medical centers participating in this study (China-Japan Friendship Hospital, Zhengzhou Hospital of Traditional Chinese Medicine, and Beijing Anzhen Hospital), all of which will provide original Case Report Form (CRF) tables to the central QSG office at Beijing University of Chinese Medicine.

## Recruitment

Through advertisements and referrals, a total of 200 patients who qualify will be recruited at the three centers participating in this study. A principle investigator, assisted by a well-trained study coordinator in each participating center, will identify potentially eligible patients based on the eligibility criteria. And, in collaboration with the attending physician, they will confirm the patient eligibility for the trial. The study coordinator will then obtain written informed consent from the patient, which will be obtained from participating clinicians before or at the time of patient consent.

### Eligibility, exclusion, and withdrawal criteria

The eligibility criteria are as follows: (1) age between 18 and 75 years, (2) clinical findings of CHF for at least 3 months prior to screening, (3) CHF caused by coronary heart disease and hypertension, which is diagnosed according to the Chinese guidelines published in 2014 [10] for the diagnosis and treatment of heart failure, (4) in a clinically stable condition with a New York Heart Association (NYHA) functional class of II to III and an optimal medical treatment with a fixed dosage for at least 2 weeks, (5) a documented left ventricular ejection fraction (LVEF) ≤ 40% and a serum *N*-terminal pro-B-type natriuretic peptide (NT-proBNP) level ≥ 450 pg/ml, (6) CHF of *Qi* deficiency and blood stasis syndrome based on TCM syndrome differentiation, and (7) provision of written informed consent.

The exclusion criteria are stated as below: (1) CHF accompanied by severe valvular heart disease, congenital heart disease, pericardial disease, cardiomyopathy, unstable angina, acute myocardial infarction (in the recent 4 weeks), cardiogenic shock, acute myocarditis, infective endocarditis, or uncontrolled severe cardiac arrhythmia with hemodynamic changes; (2) pulmonary heart disease, pulmonary hypertension caused by acute or chronic pulmonary embolism or cerebral apoplexy in the last 6 months; (3) severe hepatic inadequacy with alanine aminotransferase or alkaline phosphatase levels more than twice the upper normal limit, renal inadequacy with a serum creatinine level > 3 mg/dl (>265 μmol/L), severe electrolyte imbalance, severe hematologic disease, malignant tumor, diabetes mellitus with severe complications, or severe endocrine diseases such as hyperthyroidism and hypothyroidism; (4) acute infection confirmed by any one of the following three indicators: (a) fever, (b) a white blood cell count > 10 × 10^9^/L and a percentage of neutrophils > 75%, (c) shadow on chest X-ray, (d) uncontrolled blood pressure or fibrosis in other organs, (e) CHF of *yin* deficiency according to TCM syndrome differentiation, (f) pregnancy or breastfeeding, (g) psychiatric or infectious disease, and (h) patients who have participated in other clinical trials in the past 2 months.

The rejection and withdrawal criteria are listed as below: (1) unsuitability as judged by the investigators, (2) participants not following the protocol, (3) no visit record after treatment, and (4) voluntary withdrawal from the trial without any available data. These data will not be excluded from the study, instead, they will fall within ITT (intention-to-treat) through discussions among investigators, statisticians, data management, and others before un-blinding.

### Ethics

This trial has been authorized by the Institutional Review Board of Beijing University of Chinese Medicine (approval number: 2017 BZHYL0101). The research team will ensure this study be conducted in accordance with the principles of the Declaration of Helsinki [11] and the principles of Good Clinical Practice [12]. Signed informed Consent Forms will be obtained from all qualified participants before enrollment.

## Randomization

An independent statistician who is unaware of the design and purpose of the study will generate a randomization table by using Statistical Analysis System (SAS, version 9.4). The 200 patients will be randomly allocated in a 1:1 ratio to either the QSG treatment group or the placebo group. The randomization will be stratified by study site. According to the design of the random program, the researcher will open one random envelope and obtain a randomization number for a patient on the day of inclusion. Both investigators and participants will be blind to the allocations.

## Blinding

Participants, investigators, and the clinical trial pharmacist will be blinded to the treatment allocation throughout the course of the study. The placebo granules will be similar to the QSG granules in appearance, taste, and smell. The manufacturer will label the random codes on the packaging, and the code labeling will conform to the principles of GCP. The clinical trial pharmacist at each center will provide the packaged drugs to the participants according to the randomization number. The statistician will uncover the blinding when necessary, in case a serious adverse event (SAE) occurs, which includes death, life-threatening, hospitalization, prolonged hospitalization, or causing a permanent disability, cancer, birth defects, and drug overdose.

## Intervention

### QSG

The QSG treatment group will receive *Qishen* granules (13.6 g/pouch, twice per day—30 min after breakfast and dinner—for 12 weeks; dosage based on the requirements of Pharmacopoeia of the People’s Republic of China). The *Qishen* granules are manufactured by Beijing Tcmages Pharmaceutical Co., Ltd. (Beijing, China), a company that has obtained Chinese Good Manufacturing Practice for Pharmaceutical Products certification. All the ingredients have been approved by the Chinese Ministry of Food and Drug Safety. The 13.6 g (net weight) of granules (dehydrated ingredients combined with dextrin and caramel) are composed of six herbs: *Astragalus membranaceus* (Fisch.) Bge. var. *mongolicus* (Bge.) Hsiao (6 g), *Salvia miltiorrhiza* Bge. (1.5 g), *Lonicera japonica* Thunb. (2 g), *Scrophularia ningpoensis* Hemsl. (2 g), *Aconitum carmichaeli* Debx. (0.9 g), and *Glycyrrhiza uralensis* Fisch. (1.2 g).

### Placebo

The placebo group will receive placebo granules (13.6 g/pouch, twice per day—30 min after breakfast and dinner—for 12 weeks). The placebo is also manufactured by Beijing Tcmages Pharmaceutical Co., Ltd. (Beijing, China) by conforming to the specification for processing Chinese medicine in Beijing. The placebo granules are similar to the *Qishen* granules in appearance, taste, and smell.

All products will be packaged by Beijing Tcmages Pharmaceutical Co., Ltd. (Beijing, China). Either QSG or placebo pouches will be provided to each randomized participant at visit 1 (week 0 ± 3 days), visit 2 (week 4 ± 3 days), and visit 3 (week 8 ± 3 days).

### Concomitant and forbidden drugs

Drugs recommended in the 2014 Chinese guidelines [10] for the diagnosis and treatment of heart failure are permitted. They include angiotensin-converting enzyme inhibitors (ACEIs) or angiotensin-receptor blockers (ARBs), β-blockers, diuretics, aldosterone-receptor antagonists (AAs), digoxin, and ivabradine. However, in the course of the study, the patient’s treatment is required to remain stable as far as possible. If the patient is in a serious condition and the treatment plan has to be adjusted, the clinician should operate according to the guidelines and inform the researcher of relevant information in an accurate and detailed manner. Other traditional Chinese medicines that may alleviate CHF—whether it is a decoction or a Chinese patent medicine—are prohibited. Drugs that are not related to CHF symptoms are permitted. The name, duration, and dosage of any other drugs taken will be recorded in the CRF.

## Outcome measures

### Laboratory tests

Routine laboratory tests (complete blood count, routine urinalysis, and serum chemistry profile) will be performed in the local laboratories of the participating institutions. Serum NT-proBNP levels will be measured by using an NT-proBNP (human) ELISA kit in the Research Center, Beijing University of Chinese Medicine, Beijing, China.

### Primary outcome

The primary outcome is the proportion of patients in the QSG group, compared with the placebo group, demonstrating a more than 30% decrease in NT-proBNP level during 12 weeks of treatment.

On the day of inclusion, serum NT-proBNP will be taken as the baseline level of participants who will be enrolled and randomized for this trial before taking the QSG or placebo.

### Secondary outcomes

The secondary outcomes of this study are as follows:

1. Composite cardiac events (CCEs): CCEs are defined as death, readmission for heart failure and other cardiovascular events such as acute coronary syndrome, serious cardiac arrhythmias, cardiac shock, etc. We will carry out continuous monitoring of CCEs and evaluate the links between CCEs and test drugs. The assessments of safety and tolerability will be based on spontaneous reports of adverse events, vital signs and laboratory tests
2. NYHA functional classification: based on the NYHA functional classification, the investigators will grade the severity of functional limitation and patient estimates of how far they are able to walk before they become breathless
3. 6-minute walking distance (6MWD): participants will be instructed to walk as far as possible over a period of 6 min. This test is scored in rounded meters walked in 6 min
4. LVEF: left ventricular ejection will be obtained from all participants by an experienced echocardiographic technician in each local participating center, who is blind to the allocation of the patients
5. Patient quality of life: The Chronic Heart Failure Quality of Life Scale of Integrated Chinese and Western Medicine [13] will be used to evaluate all patients. An additional file shows this in more detail (see Additional file 2)
6. TCM syndrome integral scale: TCM symptoms of all patients will be assessed through TCM syndrome integral scale

## Data collection and management

**Fig. 2:**
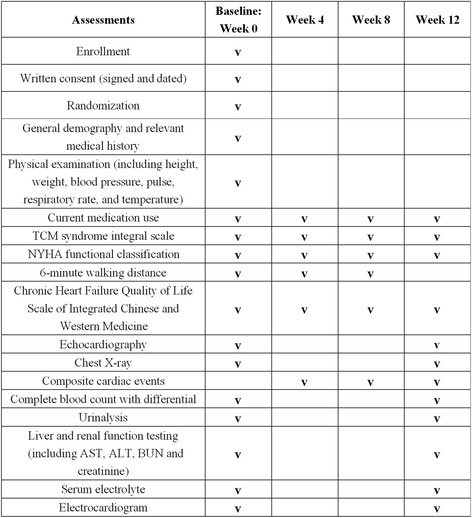
Standard Protocol Items: Recommendations for Interventional Trials (SPIRIT) flowchart

A CRF will be used for each participant to collect relevant data. The participants will be evaluated every 4 weeks during the study. Data on CCEs, NYHA functional classification, 6MWD, patient quality of life and the TCM syndrome integral scale will be collected at each visit (week 0 ± 3 days, week 4 ± 3 days, week 8 ± 3 days, and week 12 ± 3 days). The NT-proBNP and LVEF will be measured only at the first and the last visit (week 0 ± 3 days and week 12 ± 3 days). The Standard Protocol Items: Recommendations for Interventional Trials (SPIRIT) flowchart of the trial can be found in Fig. 2.

All documents collected in this study will be stored safely. Apart from the informed consent, in all other documents in this study, participants will be referred to by a specific randomization number rather than by their names. The study documentation will be archived for at least 5 years after the study.

## Adverse events (AEs)

An undesirable, unexpected sign, symptom, or disease that occurs during clinical observation will be identified as an adverse event, whether or not it has a causal relationship with the test drug. We will carry out continuous monitoring of AEs and evaluate the links between AEs and test drugs. The chief investigator will be informed of any serious AEs immediately and will cooperate with clinicians in dealing with AEs in a timely manner to protect the subjects as much as possible.

All treatment-related serious adverse events will be recorded and reported to the Research Ethics Committee in good time. The chief investigator will be responsible for all AE reporting. Prior to study initiation, all investigators involved in the study will be trained in the procedures to be followed and in the forms to be used during the study.

## Statistical analysis

### Sample size calculation

The sample size was calculated based on the expected reduction in serum NT-proBNP levels during the study. Previous studies showed that the prognosis of patients with an NT-proBNP level decrease by at least 30% from baseline through treatment is better than in those not treated [14, 15]. Therefore, according to another previous study on treating CHF with Chinese herbal medicine [4], the proportion of patients demonstrating a more than 30% decrease in NT-proBNP level in the placebo group is 31.98% (*P*_1_ = 0.3198). According to previous clinical results, the investigators assumed that the proportion of patients demonstrating a more than 30% decrease in NT-proBNP level in the QSG group would be 53% (*P*_2_ = 0.53). Given a type-I error rate of *α* = 0.05 and a type-II error rate of *β* = 0.2, and considering a dropout rate of approximately 20% among randomized patients, the investigators plan to include a total of 200 subjects. The relevant formulas are shown as follows:

$$ \mathrm{n}={\left({\mathrm{Z}}_{1-\alpha /2}+{\mathrm{Z}}_{1-\beta}\right)}^2\times \left[{\mathrm{P}}_1\left(1-{\mathrm{P}}_1\right)+{\mathrm{P}}_2\left(1-{\mathrm{P}}_2\right)\right]/{\delta}_2 $$

$$ \mathrm{N}=2{\mathrm{n}}^{\ast}\left(1+20\%\right) $$

### Data analysis

Data entry and management will be completed by an independent data administrator to ensure data accuracy. A professional statistician will perform the data analysis for the results. We will use the intent-to-treat principle to analyze the efficacy and safety of QSG. For continuous variables, the independent two-sample Student’s *t* test will be used for comparisons between the two study groups, and the paired test will be used for intra-group comparisons. The *χ*2 test will be used for categorical variables. When continuous data distribution is not normal, the Wilcoxon test will be used. *P* < 0.05 is considered to be statistically significant, and all tests are two-tailed.

## Discussion

CHF is a major worldwide health problem. Despite the medical and technological advances in treating heart failure, the mortality, morbidity and treatment costs of CHF remain high or are increasing [2]. Myocardial fibrosis plays an important role in the development of heart failure [16]. The renin-angiotensin-aldosterone system (RAAS), especially angiotensin II and aldosterone, play a key role in myocardial fibrosis [17]. Although significant progress has been made in the treatment of heart failure by ACEIs, ARBs, and AAs, recent studies have found substantial limitations in these drugs [18–20]. With its much lower cost and a good effect on CHF, TCM has been widely used in the treatment of CHF in China.

QSG is a TCM formula that has long been used to treat CHF and has good efficacy in clinical practice. Our previous study has also shown that QSG exerted cardio-protective effects and prevented left ventricular remodeling in AMI animal models [21]. Last but not least, QSG was found to have multiple targets associated with the inhibition of RAAS, thus producing cardio-protective therapeutic effects [7].

However, relevant evidence from clinical trials to show its efficacy is still scarce. The purpose of this study is to evaluate the efficacy and safety of QSG in CHF. This trial will provide high-quality evidence on the efficacy and safety of QSG in the treatment of CHF, thus providing reference for the clinical application of QSG.

### Trial status

This clinical trial was reviewed by the Medical and Laboratory Animal Ethics Committee, Beijing University of Chinese Medicine in January 2017. The study is expected to start in March 2017 and patients have not yet been recruited.

## Additional files

Additional file 1: SPIRIT Checklist. (DOC 115 kb)

Additional file 2: Chronic Heart Failure Quality of Life Scale of Integrated Chinese and Western Medicine. (DOCX 23 kb)

## Abbreviations

6MWD: 6-minute walking distance
AEs: Adverse events
CCEs: Composite cardiac events
CHF: Chronic heart failure
NT-proBNP: *N*-terminal pro-B-type natriuretic peptide
NYHA: New York Heart Association
QSG: *Qishen* granules
SPIRIT: Standard Protocol Items: Recommendations for Interventional Trials
TCM: Traditional Chinese medicine.
