# Supplementary material for: Safety and efficacy of Qishen granules in patients with chronic heart failure: study protocol for a randomized controlled trial
Source: Trials. 2017 Oct 10;18:468. doi: 10.1186/s13063-017-2193-z (PMC5634880; doi:10.1186/s13063-017-2193-z)
Supplement: Supplementary file 2 — Chronic Heart Failure Quality of Life Scale of Integrated Chinese and Western Medicine. (DOCX 23 kb) [file 13063_2017_2193_MOESM2_ESM.docx]

**Chronic Heart Failure Quality of Life Scale of Integrated Chinese and Western Medicine**

1. The following questions ask how much your heart failure (heart condition) affected your life during the past month (4 weeks).

2. After each question, circle the 0, 1, 2, 3, 4 or 5 to show how much your life was affected.

3. If a question does not apply to you, circle the 0 after that question.

4. If you had any symptome that might be related to your heart condition, then rate how much it prevented you from doing things you wanted to do or feeling the way you would like to feel. Circle either the 0, 1, 2, 3, 4, or 5 to indicate how much the symptome affected your life during the last 4 weeks – zero(0) means not at all, one(1) means very little and five(5) means very much.

| Did your heart condition prevent you from living as you wanted during the last 4 weeks by | | NO | | Very Little | |  | | | Very Much | |
| --- | --- | --- | --- | --- | --- | --- | --- | --- | --- | --- |
| **1** | causing swelling in your ankles or legs, etc.? | | 0 | 1 | 2 | | 3 | 4 | | 5 |
| **2** | giving you the feeling of suffocation? | | 0 | 1 | 2 | | 3 | 4 | | 5 |
| **3** | making you gasp for breath? | | 0 | 1 | 2 | | 3 | 4 | | 5 |
| **4** | making you short of breath? | | 0 | 1 | 2 | | 3 | 4 | | 5 |
| **5** | making your chest tightness? | | 0 | 1 | 2 | | 3 | 4 | | 5 |
| **6** | making your cough and expectoration? | | 0 | 1 | 2 | | 3 | 4 | | 5 |
| **7** | making your chest pain? | | 0 | 1 | 2 | | 3 | 4 | | 5 |
| **8** | making your appetite drop? | | 0 | 1 | 2 | | 3 | 4 | | 5 |
| **9** | making your sleeping well at night difficult? | | 0 | 1 | 2 | | 3 | 4 | | 5 |
| **10** | making your stool abnormal? | | 0 | 1 | 2 | | 3 | 4 | | 5 |
| **11** | causing your urine abnormal? | | 0 | 1 | 2 | | 3 | 4 | | 5 |
| **12** | causing your spontaneous sweating? | | 0 | 1 | 2 | | 3 | 4 | | 5 |
| **13** | causing your abdominal distension? | | 0 | 1 | 2 | | 3 | 4 | | 5 |
| **14** | causing your palpitations? | | 0 | 1 | 2 | | 3 | 4 | | 5 |
| **15** | making you feeling cold easily? | | 0 | 1 | 2 | | 3 | 4 | | 5 |
| **16** | causing your soreness and weakness of waist and knees? | | 0 | 1 | 2 | | 3 | 4 | | 5 |
| **17** | making your lips cyanotic? | | 0 | 1 | 2 | | 3 | 4 | | 5 |
| **18** | making it difficult for you to accomplish mild activities such as squat or walking 100 meters? | | 0 | 1 | 2 | | 3 | 4 | | 5 |
| **19** | making it difficult for you to take care of yourself in your daily life? | | 0 | 1 | 2 | | 3 | 4 | | 5 |
| **20** | making it difficult for you to accomplish moderate activities such as climbing one stair or walking 1000 meters? | | 0 | 1 | 2 | | 3 | 4 | | 5 |
| **21** | making it difficult for you to finish the housework? | | 0 | 1 | 2 | | 3 | 4 | | 5 |
| **22** | making you stop doing something because of physical discomfort? | | 0 | 1 | 2 | | 3 | 4 | | 5 |
| **23** | making you have to sit or lie down even during the day? | | 0 | 1 | 2 | | 3 | 4 | | 5 |
| **24** | making it difficult for you to adapt to seasonal changes? | | 0 | 1 | 2 | | 3 | 4 | | 5 |
| **25** | making you unresponsive? | | 0 | 1 | 2 | | 3 | 4 | | 5 |
| **26** | making you faint to speak? | | 0 | 1 | 2 | | 3 | 4 | | 5 |
| **27** | making you reluctant to speak because you lack strength? | | 0 | 1 | 2 | | 3 | 4 | | 5 |
| **28** | making it difficult for you to remember things? | | 0 | 1 | 2 | | 3 | 4 | | 5 |
| **29** | making you tired, fatigued, or low on energy? | | 0 | 1 | 2 | | 3 | 4 | | 5 |
| **30** | making it difficult for you to participate in normal social activities? | | 0 | 1 | 2 | | 3 | 4 | | 5 |
| **31** | making you feel you are a burden to your family or friends? | | 0 | 1 | 2 | | 3 | 4 | | 5 |
| **32** | making your recreational pastimes, sports or hobbies difficult? | | 0 | 1 | 2 | | 3 | 4 | | 5 |
| **33** | making it difficult for you to make a living? | | 0 | 1 | 2 | | 3 | 4 | | 5 |
| **34** | making it difficult for you to control your emotions? | | 0 | 1 | 2 | | 3 | 4 | | 5 |
| **35** | making you worry? | | 0 | 1 | 2 | | 3 | 4 | | 5 |
| **36** | making you feel depressed? | | 0 | 1 | 2 | | 3 | 4 | | 5 |
| **37** | costing you money for medical care? | | 0 | 1 | 2 | | 3 | 4 | | 5 |
| **38** | making you stay in a hospital? | | 0 | 1 | 2 | | 3 | 4 | | 5 |
|  |  | | Not Very  At All Much | | | | | | | |
| **Are you satisfied with your present life?** | | | 0 | 1 | 2 | | 3 | 4 | | 5 |
